# Quantitative Assessment of Landslide Susceptibility Comparing Statistical Index, Index of Entropy, and Weights of Evidence in the Shangnan Area, China

**DOI:** 10.3390/e20110868

**Published:** 2018-11-10

**Authors:** Jie Liu, Zhao Duan

**Affiliations:** 1 Propaganda Department, Shaanxi Radio and TV University, Xi’an 710119, China; 2 College of Geology & Environment, Xi’an University of Science and Technology, Xi’an 710054, China

**Keywords:** landslide susceptibility, statistical models, comparison, Shangnan County, China

## Abstract

In this study, a comparative analysis of the statistical index (SI), index of entropy (IOE) and weights of evidence (WOE) models was introduced to landslide susceptibility mapping, and the performance of the three models was validated and systematically compared. As one of the most landslide-prone areas in Shaanxi Province, China, Shangnan County was selected as the study area. Firstly, a series of reports, remote sensing images and geological maps were collected, and field surveys were carried out to prepare a landslide inventory map. A total of 348 landslides were identified in study area, and they were reclassified as a training dataset (70% = 244 landslides) and testing dataset (30% = 104 landslides) by random selection. Thirteen conditioning factors were then employed. Corresponding thematic data layers and landslide susceptibility maps were generated based on ArcGIS software. Finally, the area under the curve (AUC) values were calculated for the training dataset and the testing dataset in order to validate and compare the performance of the three models. For the training dataset, the AUC plots showed that the WOE model had the highest accuracy rate of 76.05%, followed by the SI model (74.67%) and the IOE model (71.12%). In the case of the testing dataset, the prediction accuracy rates for the SI, IOE and WOE models were 73.75%, 63.89%, and 75.10%, respectively. It can be concluded that the WOE model had the best prediction capacity for landslide susceptibility mapping in Shangnan County. The landslide susceptibility map produced by the WOE model had a profound geological and engineering significance in terms of landslide hazard prevention and control in the study area and other similar areas.

## 1. Introduction

Landslides, as one of the most critical geological hazards in the world, seriously threaten lives, property and natural resources [1,2,3,4,5]. According to the latest statistics on geological disasters carried out by the Chinese Geological Environment Information Site, more than 270,000 geological hazards occurred from 2006 to 2016, causing a direct economic loss of $7.7 billion, and the proportion of loss caused by landslides has increased year by year (http://www.cigem.gov.cn). Hence, in order to reduce the damage caused by landslides, investigating landslide susceptibility maps has become an important task that needs to be addressed [3,6,7,8,9]. Previous studies of landslide susceptibility mapping found that the quality of the data, the depth of the research and the methods of analysis were the three most important factors with a primary effect on the accuracy and reliability of the assessment results [6,9,10,11].

Along with the application of global positioning systems (GPS), remote sensing (RS), and geographic information systems (GIS) to landslide susceptibility mapping, more and more researchers have begun to apply relevant theories to landslide susceptibility assessment. These methods can be categorized into heuristic, deterministic, and statistical approaches [12]. Heuristic approaches are completely based on expert opinions or approaches, which are intensively subjective [12,13]. Deterministic approaches need a large number of detailed input factors to build models, which require field-based geotechnical and groundwater data; thus, these approaches are often used to prepare maps of small areas [12,13,14]. Therefore, statistical models are most commonly used in landslide susceptibility mapping.

Statistical models can be further categorized into traditional statistical methods, advanced machine learning technologies, and hybrid integration approaches. Traditional statistical methods are widely used, such as frequency ratio [15,16], evidential belief function [17,18,19], statistical index [13,20], weights of evidence [21,22,23], index of entropy [24,25,26,27,28], and logistic regression [29,30]. In recent decades, machine learning technologies have continuously introduced new and powerful approaches, such as naïve Bayes [31,32], naïve Bayes tree [33,34,35], artificial neural networks [16,36,37], kernel logistic regression [34,38,39], support vector machine [40,41], alternating decision tree [34,39], random forest [12,26,42,43], and multivariate adaptive regression spline [44,45]. In the interest of improving the accuracy of the prediction, some ensemble models have been proposed, such as adaptive, neuro-fuzzy inference, system–genetic algorithms [46]; bagging-based decision tree [47,48,49]; bivariate statistical-based ensembles [19,50]; adaptive neuro-fuzzy inference, system-shuffled, frog-leaping algorithms [51]; and artificial neural network-maximum entropy [52]. 

Some review articles show that different models have different characteristics, and each of them has strengths and weaknesses [41,53]. In the current research, we address compare three statistical models, applying, analyzing and inspecting the statistical index (SI), index of entropy (IOE), and weights of evidence models (WOE) with regard to landslide susceptibility mapping, using the case study of Shangnan Country, China. 

## 2. Study Area

**Figure 1:**
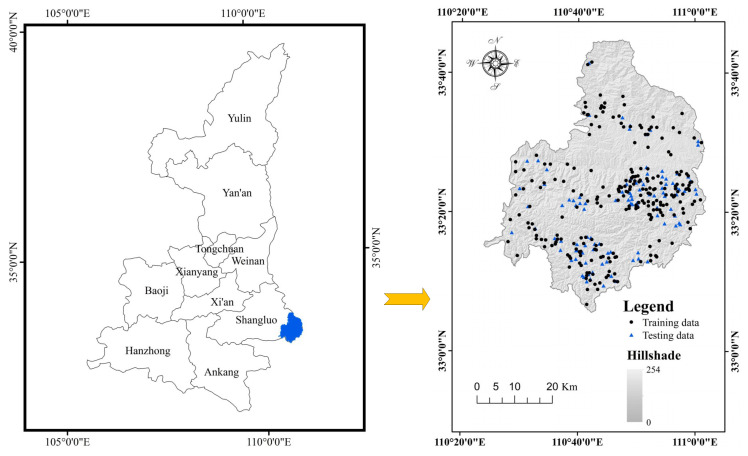
Location map of the study area.

Shaanxi Province is situated in middle of China. The study area (Shangnan County) is located in the southeastern part of Shaanxi Province, China, between the latitudes of 33°06′ and 33°44′ N, and the longitudes of 110°24′ and 111°01′ E (see Figure 1). It covers an area of about 2307 km^2^ and its altitude ranges from 189 to 2050 m above sea level. Shangnan County is located in the transitional section from the northern subtropical zone to the warm temperate zone, which is characterized by warm, abundant rainfall and four distinct seasons. A major part of the study area is covered by grassland (44.91%), followed by forestland (32.98%), farmland (21.58%), residential areas (0.41%), water bodies (0.09%), and bare land (0.02%).

Topographically, the slope gradients vary from 0° to 65°. Approximately 16.80% of the study area has a slope gradient less than 10°, whereas areas with a slope gradient larger than 50° account for 0.41% of the total study area. Areas with the slope gradient of 10–20°, 20–30°, 30–40°, and 40–50° account for 29.92%, 32.79%, 15.57%, and 4.51% of the total study area, respectively. 

Geologically, the study area is located at the border of the Yangtze and the North China plates. The faults of the study area have mainly a Northwest-Southeast direction [12]. The outcropped strata in the area are mainly from the Archaic to the Ordovician, and the Devonian and Carboniferous are partially outcropped. Since the Quaternary, the Earth’s crust has risen strongly and differential block movement has occurred. Therefore, the river is mainly characterized by down-cutting erosion, forming a deep “V” shaped valley in Danjiang River. Geomorphologically, the northern region of the study area is the middle mountainous area, the southcentral region is the middle and low mountains, while the mid-east region is faulted basin. According to the landforms, the study area could be divided into mid-mountain zones, low-relief zones, and river valley zones, with altitudes larger than 1000 m, 500–1000 m, and less than 500 m, respectively.

## 3. Data

The amount, distribution and characteristics of existing landslides were the basis of the susceptibility assessment. A landslide inventory map of a study area is effective and is organized to demonstrate the basic information regarding existing landslides [44,54]. In this case, the historical data on landslides and related information—including the topographical, geological and meteorological conditions—were acquired using three approaches, namely the analysis of existing historical records, interpretation of satellite images and field surveys in Shangnan County, respectively. In total, 348 existing landslides were identified, of which most of the landslides in the study area are slides (326), the others include 12 rock falls and 10 debris flow [12,55]. According to an analyse in the GIS environment, the size of the largest landslide is more than 30,000 m^2^, the smallest landslide is nearly 15 m^2^, while the average is 9600 m^2^. In addition, the shape and scale of the landslides in Shangnan County were simplified as a centroid point to establish the susceptibility assessment models. Finally, 348 landslides were randomly divided into training data (70%) and testing data (30%) (Figure 1).

In this paper, a total of thirteen landslide conditioning factors were employed to establish a series of mathematical models; the conditioning factors included slope angle, slope aspect, elevation, plan curvature, profile curvature, stream power index (SPI), sediment transport index (STI), topographic wetness index (TWI), distance to faults, distance to rivers, distance to roads, normalized difference vegetation index (NDVI), and lithology.

**Figure 2:**
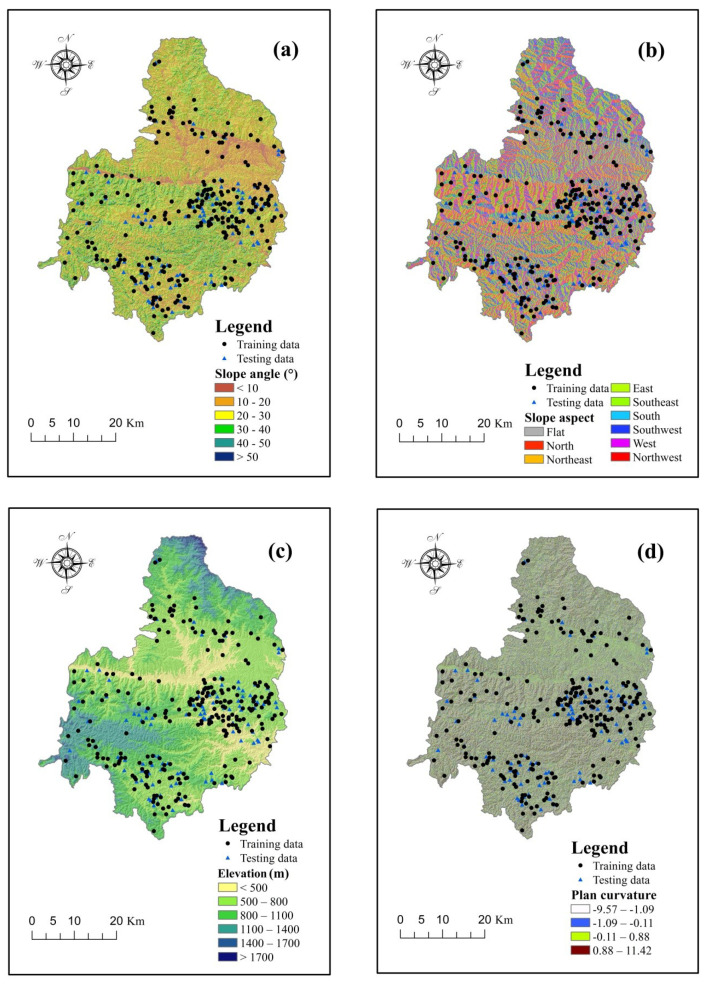

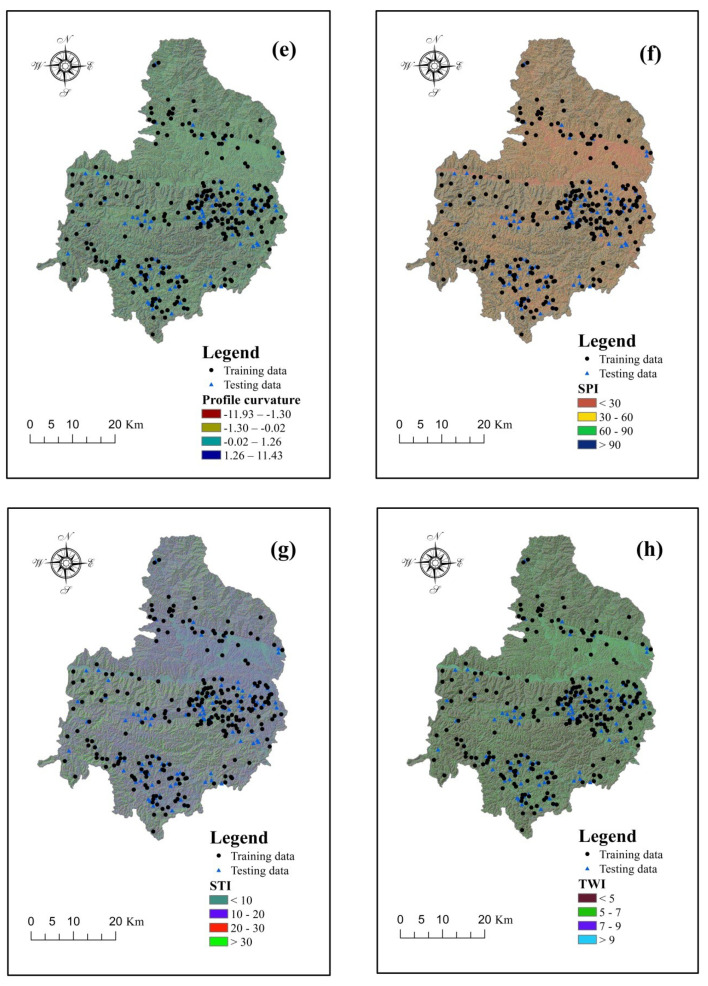

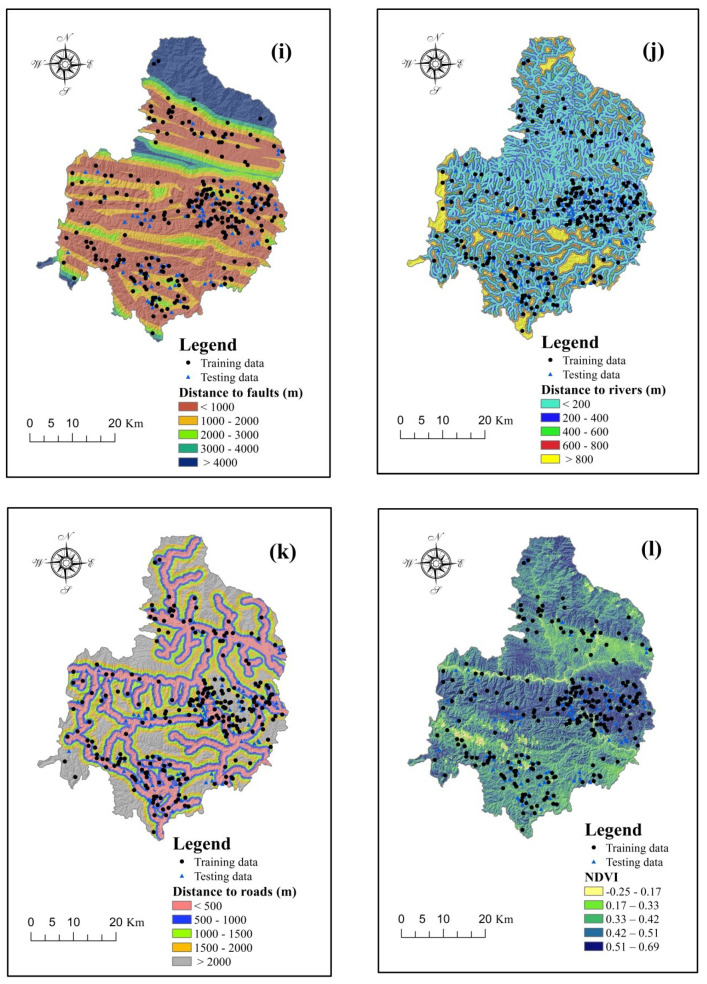

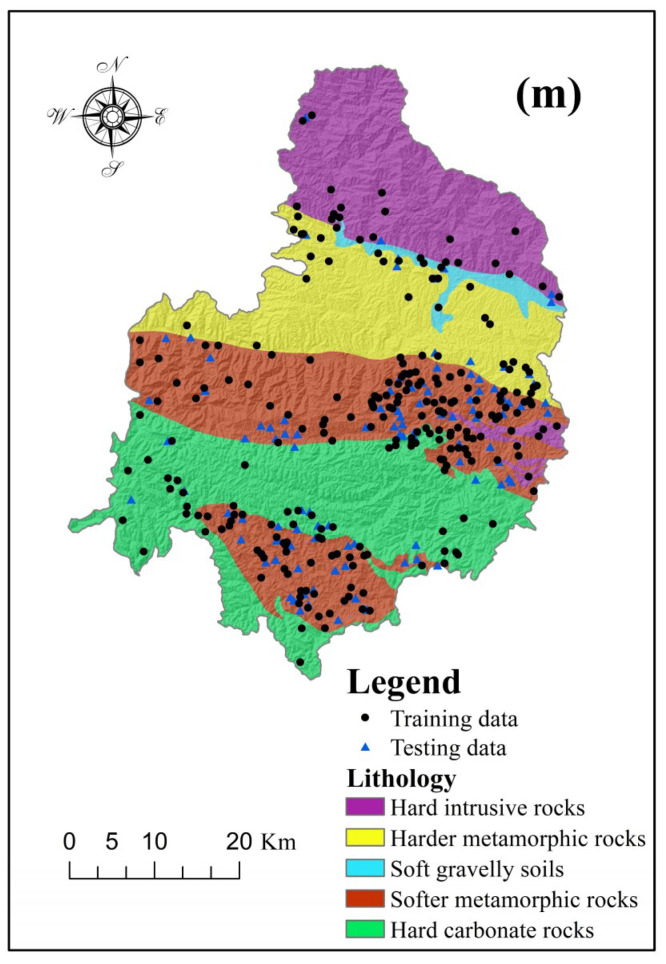
Landslide conditioning factors: (**a**) slope angle; (**b**) slope aspect; (**c**) elevation; (**d**) plan curvature; (**e**) profile curvature; (**f**) stream power index (SPI); (**g**) sediment transport index (STI); (**h**) topographic wetness index (TWI); (**i**) distance to faults; (**j**) distance to rivers; (**k**) distance to roads; (**l**) normalized difference vegetation index (NDVI); (**m**) Lithology.

Slope angle is related to the failure mode and scale of the landslide, and was used widely and frequently in landslide susceptibility assessment [31,56,57,58]. Thus, the values of the slope angles in Shangnan County were extracted from the digital elevation model (DEM) with a resolution of 30 m and divided into six categories with an interval of 10°, namely, <10°, 10–20°, 20–30°, 30–40°, 40–50°, and >50° (Figure 2a).

Slope aspect is another critical parameter used broadly in landslide susceptibility assessment. This factor can influence the meteorological conditions, such as rainfall, evaporation, temperature, etc. These meteorological conditions are generally connected to the stability of slopes [59,60]. Additionally, based on the DEM, the slope aspects in the study area were grouped into nine categories, as shown in Figure 2b.

The varieties of elevation reflect the changes in landforms between different geomorphic units. Therefore, elevation is also a relevant landslide conditioning factor used frequently in the establishment of landslide susceptibility assessment models [29,45,61]. In this study, the elevation values in Shangnan County were divided into six classes with an interval of 300 m, as follows: <500 m, 500–800 m, 800–1100 m, 1100–1400 m, 1400–1700 m, and >1700 m (Figure 2c).

Curvature, a technical term in topography, is the rate of change of the slope gradient or aspect in a particular direction [62]. Moreover, curvature can be further divided into plan curvature and profile curvature. The former is the curvature of a contour line formed by intersecting a horizontal plane with the surface, while the latter refers to the curvature in the vertical plane parallel to the slope direction [63,64]. For this reason, it was helpful to consider plan curvature and profile curvature in this study. By analyzing the DEM in the ArcGIS software (10.0, Esri, California, MA, USA), the plan curvature and profile curvature values in the study area were obtained and grouped into four classes based on the natural break method [25] (Figure 2d–e).

The stream power index (SPI) is a parameter measuring the stream power and erosion power of flowing water [65]. The scouring and infiltration of flowing water have a strong effect on the strength of the soil and rock that compose a slope. In the present study, the SPI values were arranged in four classes with an interval of 30, namely <30, 30–60, 60–90, and >90 (Figure 2f).

The sediment transport index (STI) is used to measure the erosive and transporting capacity of a stream [14]. In this study area, the STI values were divided into four categories with an interval of 10: <10, 10–20, 20–30, and >30 (Figure 2g).

The topographic wetness index (TWI) reflects the degree of accumulation of water at a site [66]. The TWI values in the study area were calculated and classified into four categories with an interval of 2 as follows: <5, 5–7, 7–9, and >9 (Figure 2h).

Generally speaking, faults can weaken the mechanical characteristics of the rock and soil of adjacent slopes [67]. Based on the ArcGIS software, buffers consisting of the Euclidean distance to faults were generated. Taking an equal interval of 1000 m, the values of the distance to faults are shown in Figure 2i, namely, <1000 m, 1000–2000 m, 2000–3000 m, 3000–4000 m, and >4000 m.

The seepage force generated by the discharge along slopes and rivers and the wetting effects of rivers have an adverse influence on the stability of slopes [68]. In this case, buffers consisting of the Euclidean distance to rivers were formed and are shown in Figure 2j. According to the equal interval classification method, there are five categories, namely <200 m, 200–400 m, 400–600 m, 600–800 m, and >800 m.

In Shangnan County, road building is one of the most major human engineering activities. Road construction frequently leads to the excavation of the toe of slopes, which may contribute to the occurrence of landslides [69]. In this case, the influence of roads was measured by the distance to roads, and the values were classified into five classes with an interval of 500 m: <500 m, 500–1000 m, 1000–1500 m, 1500–2000 m, and >2000 m, respectively (Figure 2k).

The normalized difference vegetation index (NDVI) is also universally applied in the process of landslide susceptibility assessment [25,70]. This parameter indicates the conditions of the vegetation coverage in the study area. By analyzing the near-infrared and the red band of Landsat 8 Operational Land Imager (OLI) images (http://www.gscloud.cn/), the NDVI values were calculated and classified into five classes based on the natural break method [34,71]: −0.25 to 0.17, 0.17–0.33, 0.33–0.42, 0.42–0.51, and 0.51–0.69 (Figure 2l).

Lithology is one of the most fundamental factors that determines the physical and mechanical properties of rock and soil [72,73]. Based on the field surveys and geological mapping, the lithological map of Shangnan County was digitized using the ArcGIS software. As is shown in Figure 2m, the lithological units in study area were grouped into nine categories based on the geological ages and lithofacies.

## 4. Modeling Approaches

### 4.1. Statistical Index (SI)

The statistical index model was first proposed by van Westen et al. [74]. In the SI model, a weight value for a parameter class can be defined as the natural logarithm of the landslide density in the class, divided by the landslide density in the whole study area [75,76]:

(1) $$W_{i}{}_{j}=\ln(\frac{D_{i}{}_{j}}{D})\;$$

where $W_{i}{}_{j}$ is the weight for the class i of factor j, $D_{i}{}_{j}$ is the landslide density within class i of the factor j, and D is the landslide density in the whole study area.

### 4.2. Index of Entropy (IOE)

The index of entropy is the second model used in this study. The entropy indicates the extent of the disorder of a system [77]. The equations used to calculate the information coefficient $W_{j}$ are expressed as below:

(2) $$W_{j}=I_{j}\times P_{j}\;$$

(3) Ij=Hjmax−HjHjmax,I=(0,1),j=1,2,…,n 

(4) $$H_{j}=-\sum_{i=1}^{Sj}\left(P_{ij}\right)\log_{2}(P_{ij}),j=1,2,\ldots ,n\;$$

(5) $$H_{j\max}=\log_{2}S_{j}\;$$

(6) $$(P_{ij})=\frac{P_{ij}}{\sum_{j=1}^{S_{j}}P_{ij}}\;$$

(7) $$P_{i}{}_{j}=\frac{m}{n}\;$$

where $W_{j}$ is the resultant weight value for the factors as a whole, $P_{j}$ is the slope failure probability for j=1,2,…,n, $I_{j}$ is the information coefficient, *H_j_* and *H_jmax_* are the entropy values, *S_j_* is the number of classes, and *m* and *n* are the landslide and domain percentages, respectively.

### 4.3. Weights of Evidence (WOE)

The WOE method is a probabilistic approach based on a log linear form of Bayes’ rule, expressed as:

(8) $$P(A|B)=P(B|A)\times \frac{P(A)}{P(B)}\;$$

where *A* is the presence or absence of the landslide in the study area, and *B* is the landslide predictive factor. The approach calculates the weight for each *B* based on *A,* as follows [78,79]:

(9) $$W_{i}^{+}=\ln\left(\frac{p(B|A)}{P(B|\bar{A})}\right)\;$$

(10) $$W_{i}^{-}=\ln\left(\frac{p(\bar{B}|A)}{P(\bar{B}|\bar{A})}\right)\;$$

where $W_{i}^{+}$ is an indicator of the positive correlation, $W_{i}^{-}$ shows the level of negative correlation, B is the presence of a desired class of landslide conditioning factor, and $\bar{B}$ is the absence of desired class of landslide conditioning factor. A is the presence and $\bar{A}$ is the absence of the landslide. The difference between the two weights is called the weight contrast: $C=W_{i}^{+}-W_{i}^{-}$. The contrast reflects the overall spatial correlation between the desired class of landslide conditioning factor and the landslides. 

### 4.4. Selection of Landslide Conditioning Factors

In landslide susceptibility modelling, landslides usually occur under different conditions, and the contribution of the conditioning factors to landslide occurrence is quite different [48]. Therefore, the removal of unimportant landslide conditioning factors to improve the performance of landslide models is necessary [80,81]. In this study, the SI, IOE, and WOE models were employed to construct the landslide susceptibility maps. Nevertheless, one of the most critical assumed conditions of these models is the independence assumption among the conditioning factors [38]. Therefore, in the present study, the coefficient of variation (CV) attribute evaluation (CVA) method was used to validate all thirteen landslide conditioning factors considered for the development of landslide susceptibility models. This method evaluates the worth of an attribute by computing the value of the coefficient of variation with respect to the class. It first creates a ranking of attributes based on the variation value, then divide this into two groups, using a verification method to select the best group [82].

## 5. Results and Discussion

### 5.1. Selection of Landslide Conditioning Factors

In the present study, based on the CVA method (a 10-fold cross-validation method [83,84], seed = 1), the importance of all the conditioning factors was measured according to average merit (AM), and the calculation results are illustrated in Table 1. The results show that all the AM values of the conditioning factors were larger than zero, indicating that the thirteen selected factors have positive influence on landslide occurrence. Of these factors, the highest AM value was for distance to roads (AM = 0.304), followed by elevation (AM = 0.296), distance to rivers (AM = 0.260), lithology (AM = 0.156), distance to faults (AM = 0.155), TWI (AM = 0.083), slope angle (AM = 0.069), NDVI (AM = 0.060), plan curvature (AM = 0.056), STI (AM = 0.035), slope aspect (AM = 0.032), STI (AM = 0.032), and profile curvature (AM = 0.025). Therefore, all thirteen landslide conditioning factors were selected for landslide susceptibility modeling in the present study.

### 5.2. Application of the SI Model

In this case, the SI model was applied to analyze the relationships between each conditioning factor and landslide occurrence (Table 2). From Table 2, it can be seen that for the slope angle 0–20°, the SI values were positive, which indicates that landslides were more prone to occurring in these areas. This is also in line with some other landslide susceptibility studies [81,85,86,87]. With regard to slope aspect, an eastern aspect had the highest SI value of 0.3024, while the lowest SI value was for southeast (–0.4019). In addition, no landslides occurred in flat areas (SI = 0), which conforms to actual situations and related research results [88,89]. When the altitude was lower than 800 m, there was a larger probability of landslides being triggered; all the landslides were not situated in areas with an altitude greater than 1400 m. In terms of curvature, the classes with a plan curvature of –1.09 to –0.11 (0.0124) and –0.11 to 0.88 (0.0264) had positive SI values, while the SI values were positive for classes with a profile curvature of –0.02 to 1.26 (0.0450) and 1.26 to 11.43 (0.0851). In the case of the SPI, compared with the other classes, the class of 0 to 30 (0.0801) had a more positive effect on landslide occurrence. In the case of STI, the class of >30 had the only negative SI value (–0.2712). In the case of the TWI, the intervals of 5–7 (0.0952) and 7–9 (0.1415) could be interpreted as promoting conditions. With regard to the distance to faults, the probability of landslide occurrence decreased with the increasing distance to faults, and the highest SI value of 0.2902 was for the class of 0–1000 m. For the distance to rivers, the only positive SI value of 0.2964 belonged to the class <200 m. For the distance to roads, landslides mainly spread in areas of where the distance to roads was within 500 m. Both the highest NDVI and the lowest NDVI had a positive impact on landslide occurrence. In the case of lithology, the SI values of the harder metamorphic rocks, softer metamorphic rocks, hard carbonate rocks, hard intrusive rocks and soft gravelly soils were –0.5121, 0.6650, –0.4742, –0.7160, and 0.3584, respectively.

**Figure 3:**
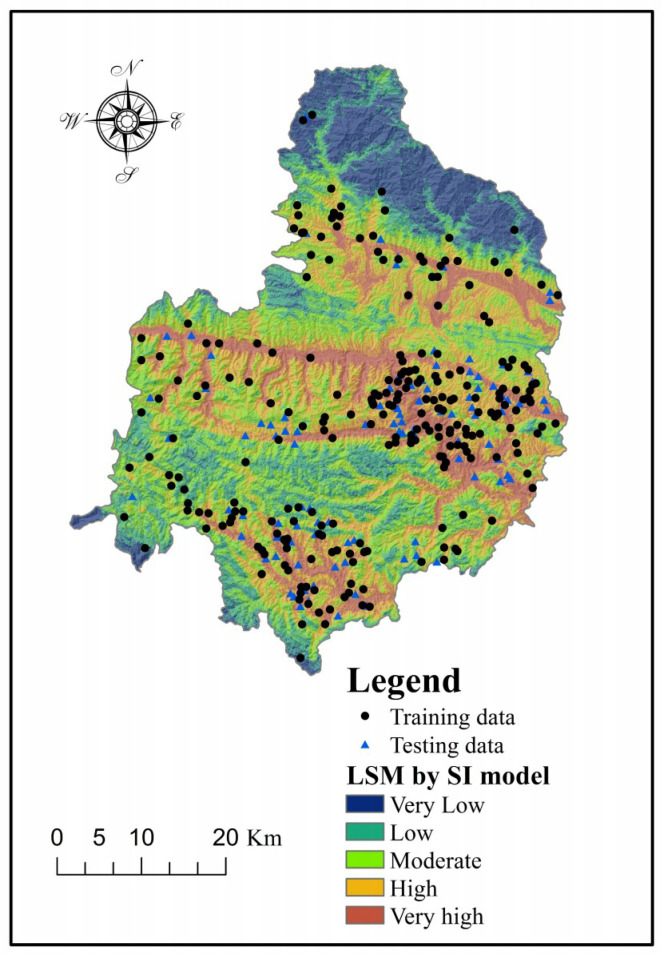
Landslide susceptibility map produced using the statistical index (SI) model.

Finally, the landslide susceptibility indexes (LSI) were calculated using the SI values and Equation (11). The corresponding landslide susceptibility map (LSM) (Figure 3) was generated using ArcGIS software. It is clear that the probability of landslide occurrence rises with the enlargement of the LSI. In the present study, the natural break method, which seeks to reduce the variance within classes and maximize the variance between classes [90], was used to the reclassify the LSI values into five categories, namely very low, low, moderate, high and very high.

(11) $$\begin{array}{l} {\mathrm{LSI}}_{\mathrm{SI}}=\mathrm{Slope}\;{\mathrm{angle}}_{\mathrm{SI}}+\mathrm{Slope}\;{\mathrm{aspect}}_{\mathrm{SI}}+{\mathrm{Elevation}}_{\mathrm{SI}}+\mathrm{Plan}\;{\mathrm{curvature}}_{\mathrm{SI}}+\mathrm{Profile}\;{\mathrm{curvature}}_{\mathrm{SI}}\\ +{\mathrm{SPI}}_{\mathrm{SI}}+{\mathrm{STI}}_{\mathrm{SI}}+{\mathrm{TWI}}_{\mathrm{SI}}+\mathrm{Distance}\;\mathrm{to}\;{\mathrm{faults}}_{\mathrm{SI}}+\mathrm{Distance}\;\mathrm{to}\;{\mathrm{rivers}}_{\mathrm{SI}}+\mathrm{Distance}\;\mathrm{to}\;{\mathrm{roads}}_{\mathrm{SI}}\\ +{\mathrm{NDVI}}_{\mathrm{SI}}+{\mathrm{Lithology}}_{\mathrm{SI}} \end{array}$$

### 5.3. Application of the IOE Model

**Figure 4:**
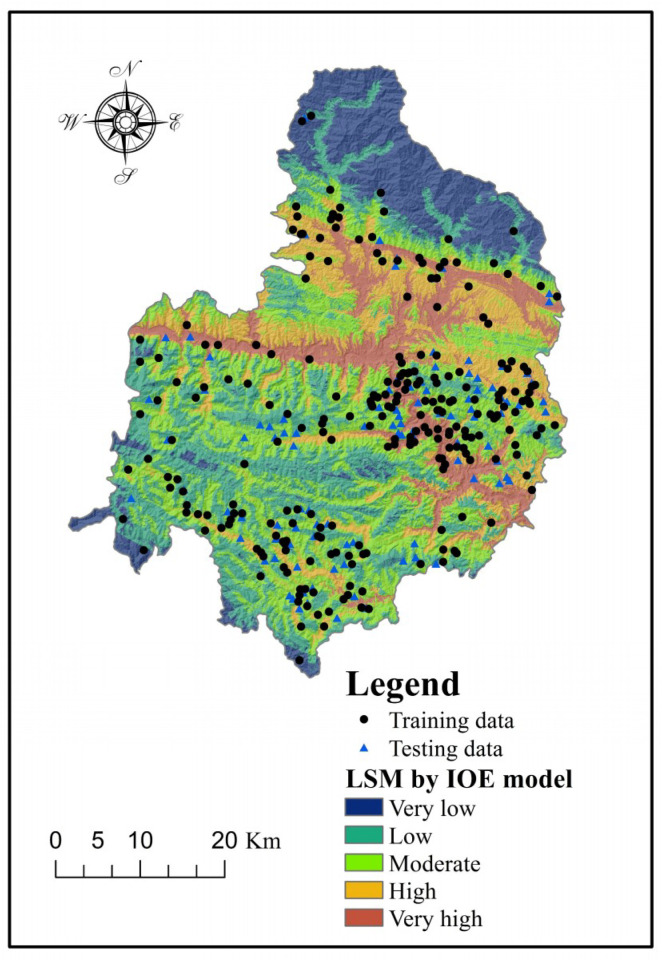
Landslide susceptibility map produced using the index of entropy (IOE) model.

From Table 2, we acquired the *W_j_* values of various conditioning factors. *W_j_* is an index to measure the importance of factors. Thus, it can be seen that the most critical factor was altitude (*W_j_* = 0.1923), followed by distance to faults (*W_j_* = 0.1068), lithology (*W_j_* = 0.0954), slope aspect (*W_j_* = 0.0560), distance to roads (*W_j_* = 0.0546), distance to rivers (*W_j_* = 0.0511), slope angle (*W_j_* = 0.0202), NDVI (*W_j_* = 0.0145), TWI (*W_j_* = 0.0090), STI (*W_j_* = 0.0063), profile curvature (*W_j_* = 0.0053), SPI (*W_j_* = 0.0022), and plan curvature (*W_j_* = 0.0006). It should be explained that the above ranking only applies to Shangnan County. The relative importance of conditioning factors usually varies for different study areas [91]. To produce a landslide susceptibility map using the LSI, the landslide occurrence probability values were calculated using Equation (12). Similarly, the produced landslide susceptibility map was further classified into five classes based on the natural break method, including very low, low, moderate, high, and very high (Figure 4).

(12) $$\begin{array}{l} {\mathrm{LSI}}_{\mathrm{IOE}}=\mathrm{Slope}\;\mathrm{angle}\times 0.0202+\mathrm{Slope}\;\mathrm{aspect}\times 0.0560+\mathrm{Elevation}\times 0.1923+\mathrm{Plan}\;\mathrm{curvature}\times 0.0006\\ +\mathrm{Profile}\;\mathrm{curvature}\times 0.0053+\mathrm{SPI}\times 0.0022+\mathrm{STI}\times 0.0063+\mathrm{TWI}\times 0.0090\;\\ +\mathrm{Distance}\;\mathrm{to}\;\mathrm{roads}\times 0.0546+\mathrm{NDVI}\times 0.0145+\mathrm{Lithology}\times 0.0954 \end{array}$$

### 5.4. Application of the WOE Model

In Table 2, the weight contrast values are noted as *C*, which indicate the landslide susceptibility of various classes of conditioning factors. In terms of the slope angle, landslides are more likely to occur in areas with a slope angle of 0–10° (0.273) and 20–30° (0.029). For slope aspect, east (0.244) had the highest probability of triggering landslides, which is in line with the conclusion of the SI model. For altitude, most landslides are more prone to occurring at altitudes <800 m. For curvature, the results showed that the highest contrast value (0.130) was for plan curvatures between –0.11 and 0.88, while profile curvatures from –0.02 to 1.26 (0.108) were most prone to landslides. For the SPI, the WOE results were the same as the SI results, and the class 0–30 had the highest contrast value of 0.264. For the STI, class of 10–20 had the only positive value (0.177), which indicates that areas with STI values of 10–20 had a positive effect on landslide occurrence. For TWI, the highest contrast value (0.235) was found for class <5. In the case of distance to faults, distance to rivers and distance to roads, the highest contrast values belonged to the class <1000 m for distance to faults, the class <200 m for distance to rivers, and the class <500 m for distance to roads. For the NDVI, it was found that the range 0.17–0.33 was the only class for which the contrast value was larger than zero. In the case of lithology, hard carbonate rocks and hard intrusive rocks were identified as promoting landslides, this result did not coincide with the results of the SI model.

**Figure 5:**
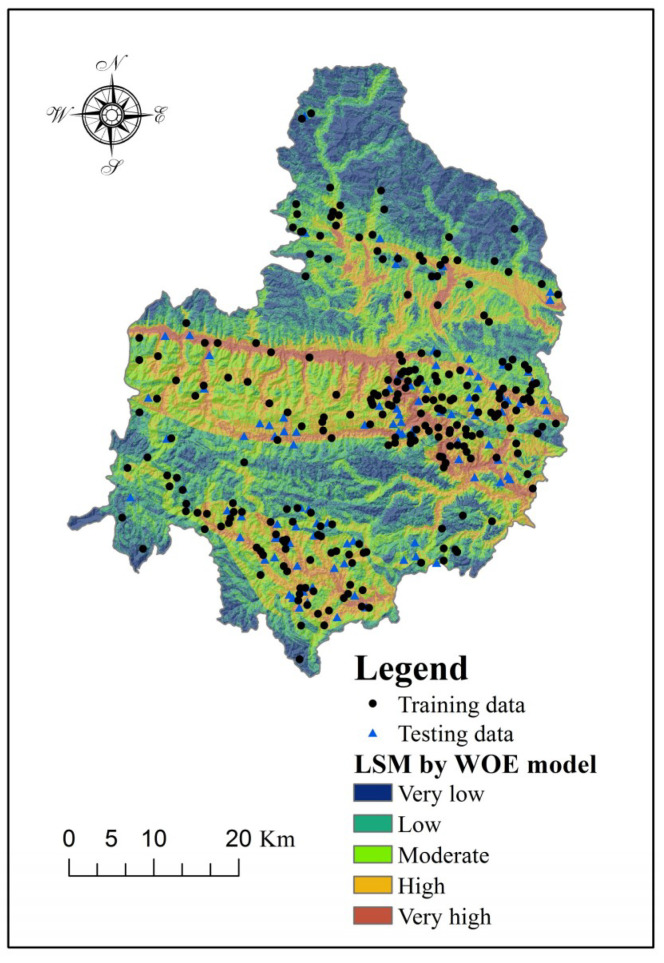
Landslide susceptibility map produced using the weights of evidence (WOE) model.

Finally, based on the results of the WOE model, the LSI values for the study area were calculated using Equation (13). The natural break method was introduced to reclassify landslide susceptibility into five classes: very low, low, moderate, high, and very high (Figure 5):

(13) $$\begin{array}{l} {\mathrm{LSI}}_{\mathrm{WOE}}=\mathrm{Slope}\;{\mathrm{angle}}_{C/S(C)}+\mathrm{Slope}\;{\mathrm{aspec}}_{C/S(C)}+{\mathrm{Elevation}}_{C/S(C)}+\mathrm{Plan}\;{\mathrm{curvature}}_{C/S(C)}\\ +\mathrm{Profile}\;{\mathrm{curvature}}_{C/S(C)}+{\mathrm{SPI}}_{C/S(C)}+{\mathrm{STI}}_{C/S(C)}+{\mathrm{TWI}}_{C/S(C)}+\mathrm{Distance}\;\mathrm{to}\;{\mathrm{faults}}_{C/S(C)}\\ +\mathrm{Distance}\;\mathrm{to}\;{\mathrm{rivers}}_{C/S(C)}+\mathrm{Distance}\;\mathrm{to}\;{\mathrm{roads}}_{C/S(C)}\;+{\mathrm{NDVI}}_{C/S(C)}+{\mathrm{Lithology}}_{C/S(C)} \end{array}$$

### 5.5. Validation and Comparison of the Models

It is absolutely necessary to quantitatively measure the accuracy of the landslide susceptibility maps produced by the various classification models [92]. To assess the performance of the three landslide susceptibility mapping models described above, the corresponding area under the curve (AUC) curves for the training dataset and testing dataset were obtained. The receiver operating characteristics (ROC) curve and the AUC are two common indices used in the validation and comparison of different landslide susceptibility models [33,34,51,80,93]. In the present study, the AUC method, which was plotted using the cumulative area percentages as the horizontal axis and the cumulative percentage of landslides as the longitudinal axis [17,19,94], was used to compare the performance of the three models. Generally, the model with the highest AUC value was considered to show the best landslide susceptibility mapping performance. 

**Figure 6:**
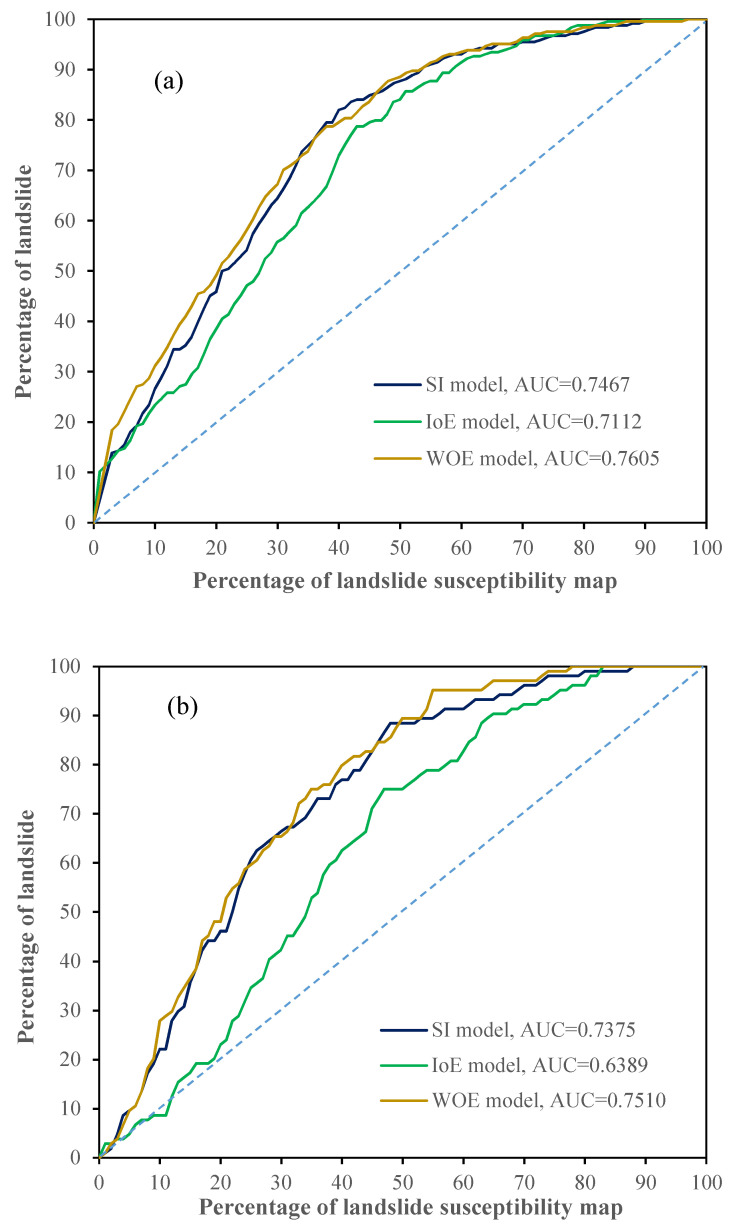
Area under the curve (AUC) curves of the models using (**a**) training data and (**b**) testing data.

In the case of the training dataset, the AUC values for the SI, IOE, and WOE models were 0.7467, 0.7112, and 0.7650, respectively, and the corresponding accuracy rates were 74.67%, 71.12%, and 76.50% (Figure 6a). It was clear that the landslide susceptibility map generated with the WOE model was more in line with actual situations. The performance of the SI model was second only to the WOE model. Compared with the other models, the accuracy of the IoE model was relatively low.

In the case of the testing dataset, the prediction accuracy values for the SI, IOE and WOE models were 73.75%, 63.89% and 75.10%, respectively (Figure 6b). The results showed that WOE had the best prediction capacity, followed by the SI model and the IOE model. In addition, the AUC values of the testing dataset were lower than those of the training dataset. When using the IOE model, the AUC value calculated with the testing dataset decreased by 0.0723 compared to the results found using the training dataset. Therefore, it could be concluded that the landslide susceptibility maps produced by the SI and WOE models both had good spatial effectiveness for the study area, and that the IoE model was not very suitable for landslide susceptibility mapping in Shangnan County.

## 6. Conclusions

In recent years, understanding of the serious effects of landslides on people’s life and property has increased. Thus, it is necessary to promote landslide susceptibility assessment in landslide hazard zones. Classical probability models and novel machine learning algorithms should be introduced to landslide susceptibility modeling with the aim of acquiring better prediction accuracy.

In this paper, the SI, IOE, and WOE models were employed to assess landslide susceptibility in Shangnan County, and the performance of the three models was compared. According to their relevance and suitability, thirteen conditioning factors were selected for modeling. The landslide data were then classified into two groups, namely a training dataset (70% of the landslides) and a testing dataset (30% of the landslides). The importance of the conditioning factors was also evaluated using the CVA method with AM values. The results showed that all the thirteen conditioning factors had a positive effect on landslide occurrence. The AUC plots generated with training dataset demonstrated that the WOE model (AUC = 0.7605) had the highest accuracy of landslide susceptibility mapping, followed by the SI model (AUC = 0.7467) and IOE model (AUC = 0.7112). Similarly, the prediction capacity of the three models was measured using AUC plots generated from the testing dataset. The results indicated that the WOE model had the best performance in landslide susceptibility prediction. 

The landslide susceptibility map produced by the WOE model can be meaningful for landslide hazard prevention and control in Shangnan County and other mountainous areas with similar features. The landslide susceptibility maps can also be used as a basis for future landslide risk assessment studies of the study area and other areas with similar geo-environmental characteristics. The model can also be applied in other areas to expand its use.

## Figures and Tables

**Table 1:** Importance of conditioning factors based on the coefficient of variation attribute (CVA) method.

Landslide Conditioning Factors	Average Merit (AM)	Standard Deviation (SD)
Distance to roads	0.304	±0.021
Elevation	0.296	±0.027
Distance to rivers	0.260	±0.016
Lithology	0.156	±0.021
Distance to faults	0.155	±0.021
TWI ^1^	0.083	±0.029
Slope angle	0.069	±0.027
NDVI ^2^	0.060	±0.026
Plan curvature	0.056	±0.026
SPI ^3^	0.035	±0.015
Slope aspect	0.032	±0.009
STI ^4^	0.032	±0.022
Profile curvature	0.025	±0.027

^1^ Topographic wetness index (TWI); ^2^ Normalized difference vegetation index (NDVI); ^3^ Stream power index (SPI); ^4^ Sediment transport index (STI).

**Table 2:** Correlation between landslides and conditioning factors using the statistical index (SI), index of entropy (IOE), and weights of evidence (WOE) models.

Conditioning Factors	Classes	No. of Pixels	No. of Landslide	SI	W*_j_*	C
Slope angle (°)	0–10	347,597	40	0.2023	0.0202	0.273
	10–20	778,722	87	0.1728	-	–0.001
	20–30	833,307	67	–0.1562	-	0.029
	30–40	489,019	43	–0.0667	-	–0.234
	40–50	135,021	6	–0.7492	-	–0.155
	50–65	12,201	1	–0.1370	-	–0.143
Slope aspect	Flat	167	0	0.000	0.0560	0.000
	North	300,994	27	–0.0467	-	0.028
	Northeast	318,751	32	0.0658	-	0.213
	East	345,947	44	0.3024	-	0.244
	Southeast	349,816	22	–0.4019	-	–0.309
	South	312,150	34	0.1474	-	0.170
	Southwest	333,799	24	–0.2680	-	–0.503
	West	320,387	31	0.0290	-	0.104
	Northwest	313,856	30	0.0168	-	–0.101
Altitude (m)	<500	345,079	42	0.2584	0.1923	0.331
	500–800	1,167,240	161	0.3835	-	0.842
	800–1100	688,633	32	–0.7045	-	–0.837
	1100–1400	350,308	9	–1.2971	-	–1.656
	1400–1700	38,955	0	0.000	-	–1.287
	>2050	5652	0	0.000	-	0.000
Plan curvature	–9.57 to –1.09	265,054	23	–0.0799	0.0006	–0.043
	–1.09 to –0.11	872,136	83	0.0124	-	–0.036
	–0.11 to 0.88	1,046,545	101	0.0264	-	0.130
	0.88–11.42	412,132	37	-0.0459	-	–0.155
Profile curvature	–11.93 to –1.30	285,207	21	–0.2442	0.0053	–0.216
	–1.30 to –0.02	950,381	88	–0.0150	-	–0.060
	–0.02 to –1.26	1,047,612	103	0.0450	-	0.108
	1.26–11.43	312,667	32	0.0851	-	0.064
SPI	0–30	1,256,999	128	0.0801	0.0022	0.264
	30–60	499,682	44	–0.0653	-	–0.355
	60–90	243,080	23	0.0066	-	0.053
	>90	596,106	49	–0.1341	-	–0.123
STI	0–10	963,339	99	0.0892	0.0063	–0.137
	10–20	723,470	70	0.0289	-	0.177
	20–30	392,792	38	0.0288	-	–0.059
	>30	516,266	37	–0.2712	-	–0.149
TWI	<5	1,095,483	91	–0.1236	0.0090	0.235
	5–7	1,131,652	117	0.0952	-	–0.171
	7–9	258,581	28	0.1415	-	0.031
	>9	110,151	8	–0.2579	-	–0.180
Distance to faults (m)	0–1000	1,353,065	170	0.2902	0.1068	0.739
	1000–2000	596,473	55	–0.0192	-	–0.025
	2000–3000	219,389	13	–0.4614	-	–0.486
	3000–4000	99,497	2	–1.5425	-	–1.564
	>4000	327,420	4	–2.0405	-	–2.151
Distance to rivers (m)	<200	1,099,425	139	0.2964	0.0511	0.530
	200–400	770,613	72	–0.0060	-	0.065
	400–600	405,048	19	–0.6951	-	–0.781
	600–800	163,814	6	–0.9425	-	–1.160
	>800	156,944	8	–0.6119	--	–0.606
Distance to roads (m)	<500	696,109	113	0.5464	0.0546	0.850
	500–1000	547,849	42	–0.2038	-	–0.228
	1000–1500	439,072	39	–0.0566	-	–0.102
	1500–2000	334,495	17	–0.6149	-	–0.681
	>2000	578,319	33	–0.4991	-	–0.590
NDVI	–0.23 to 0.17	64,496	8	0.2773	0.0145	–0.439
	0.17–0.33	232,432	17	–0.2509	-	0.107
	0.33–0.42	713,855	66	–0.0165	-	–0.081
	0.42–0.51	965,450	78	–0.1514	-	–0.028
	0.51–0.71	619,630	75	0.2529	-	0.106
Lithology	Harder metamorphic rocks	514,860	29	–0.5121	0.0954	–0.824
	Softer metamorphic rocks	771,447	141	0.6650	-	–0.606
	Hard carbonate rocks	734,975	43	–0.4742	-	0.361
	Hard intrusive rocks	522,464	24	–0.7160	-	1.159
	Soft gravelly soils	52,038	7	0.3584	-	–0.607
